# Effect of the Amount of Polysorbate 80 and Oregano Essential Oil on the Emulsion Stability and Characterization Properties of Sodium Alginate Microcapsules

**DOI:** 10.3390/molecules26206304

**Published:** 2021-10-19

**Authors:** Juste Baranauskaite, Mehmet Ali Ockun, Burcu Uner, Cetin Tas, Liudas Ivanauskas

**Affiliations:** 1 Department of Pharmaceutical Technology, Faculty of Pharmacy, Yeditepe University, Kayisdagi Cd., Atasehir, Istanbul 34755, Turkey; baranauskaite.juste@gmail.com (J.B.); uner.burcu@yahoo.com (B.U.); cetin.tas@yeditepe.edu.tr (C.T.); 2 Department of Pharmacognosy, Faculty of Pharmacy, Yeditepe University, Kayisdagi Cd., Atasehir, Istanbul 34755, Turkey; mehmet.ockun@yeditepe.edu.tr; 3 Department of Analytical and Toxicological Chemistry, Faculty of Pharmacy, Medical Academy, Lithuanian University of Health Sciences, 13 Sukileliu Str., LT-50161 Kaunas, Lithuania

**Keywords:** carvacrol, extrusion, GC/MS, HPTLC

## Abstract

Essential oils have a high volatility that leads to evaporation and loss of their pharmacological effect when exposed to the environment. The objectives of the present work were to prepare microcapsules with oregano essential oil by extrusion using sodium alginate as a shell material and non-ionic surfactant polysorbate 80 as an emulsifier to stabilize the emulsion. The present study was aimed to evaluate the physical parameters of microcapsules and to compare the influence of the amount of emulsifier and the essential oil-to-emulsifier ratio on the capsules’ physical parameters and encapsulation efficiency; to our knowledge, the existing research had not yet revealed whether unstable emulsion affects the encapsulation efficiency of oregano essential oil. This study showed that increasing the emulsifier amount in the formulation significantly influenced encapsulation efficiency and particle size. Moreover, increasing the emulsion stability positively influenced the encapsulation efficiency. The emulsion creaming index depended on the emulsifier amount in the formulation: the highest creaming index (%) was obtained with the highest amount of polysorbate 80. However, the essential oil-to-polysorbate 80 ratio and essential oil amount did not affect the hardness of the microcapsules (*p* > 0.05). In conclusion, the obtained results could be promising information for production of microcapsules. Despite the fact that microencapsulation of essential oils is a promising and extremely attractive application area for the pharmaceutical industry, further basic research needs to be carried out.

## 1. Introduction

*Origanum* L., which belongs to the Lamiaceae family, is an annual, perennial, or shrubby plant [1]. There are 57 species of oregano that grow naturally in the world [2]. In Turkey, the oregano known as “mercanköşk”, is represented by 27 species and 30 taxa, 15 of which are endemic [2]. The main components in the structure of oregano oil are thymol and carvacrol. Depending on the species and growing conditions, one or both of these compounds can be present in high amounts in oregano species [3,4,5,6]. For this reason, the chemical content of the oil is important for its effectiveness [7]. Oregano essential oil has powerful health benefits. The antispasmodic, analgesic, diaphoretic, carminative, antioxidant, antibacterial, and antifungal activities of oregano essential oils have been extensively studied. Carvacrol has been identified as the main active compound responsible for these properties [8]. The volatility of essential oils limits their application as food supplements, as they are unstable and have solubility problems and require encapsulation. It is important to protect the active ingredients of essential oils in order to not lose their biological efficiency.

Microencapsulation is a process of enclosing the substances within an inert material, which protects the substances from the environment. The microcapsules can release their contents at controlled rates under specific conditions [9]. The active substances located in the core of the microcapsules are protected by a shell membrane composed of excipients in the formulation. This shell protects the essential oils from moisture, oxygen, and temperature changes, thereby extending their shelf life [10,11]. A variety of different methods are used to prepare microcapsules. The microencapsulation methods can be divided into two main groups: chemical and physical. The chemical methods include polymerization, suspension, in-situ emulsion, and dispersion; while the physical methods include spray-drying, coacervation, pan coating, extrusion, and others [12,13,14]. When using essential oils as a core material, choosing the right encapsulation technique is challenging due to its physical state. Oils are in liquid form, so the method of microencapsulation must be selected with specific properties [15]. In terms of the physical microencapsulation methods, extrusion is suitable for essential oils encapsulation. This method is described as an action of pushing out the liquids [16] using different types of devices for formation of microcapsules [17]. For example, a medical syringe with a needle or a syringe pump can be used as a tool for the extrusion process [18,19].

Deciding on the most appropriate shell material is an important step in developing microcapsules. According to the scientific literature, when the core is an herbal-based material, it is important to recognize the possibility of interactions between the core and shell materials. One approach to solve this problem is to select natural polymers as shell materials. The most commonly used polymers that constitute the shell structure are gelatin, collagen, chitosan, and sodium alginate [20,21]. Recently, there has been an increased popularity in using sodium alginate in the microencapsulation process due to its unique properties. It is a natural polysaccharide that is chemically stable and can form a strong gel barrier in the presence of water [22]. Generally, it is used with a calcium chloride solution (a cross linking agent) to form a shell structure for the microcapsules that is acceptably hard [21,22,23].

During the microencapsulation process, the emulsion properties play an important role in microcapsule quality. The emulsification step is a one of the most critical stages, because it can influence the emulsion droplet stability, prepared microcapsule size and morphology, and subsequent oregano essential oil encapsulation efficiency. The parameters influencing this step have been studied extensively [24,25]. Surface-active materials such as stabilizers and emulsifying agents are added to essential oil emulsions to prevent emulsions from layering out and separating into phases [25]. Sodium alginate has a poor emulsifying capacity [12], and to prepare a stable emulsion an emulsifier has to be incorporated in the formulation. In this research, polysorbate 80 was chosen as an emulsifier. It is a non-ionic surfactant and has a high hydrophilic–lipophilic balance (HLB) value of 15 and has a high emulsification capacity. Moreover, this emulsifier consists of a polysorbate head group, a hydrophilic moiety, and an oleyol chain as hydrophobic tail. It has frequently been used to produce emulsions containing essential oils because of its low-toxicity and cost, biocompatibility, and environmental friendliness. Furthermore, it is expected to efficiency maintain the stability of emulsions by steric stabilization in the presence of salt and at acidic pH values [26].

The two main purposes of the present work were to prepare microcapsules with oregano essential oil by the extrusion method using sodium alginate as a shell material and polysorbate 80 as a non-ionic surfactant and to determine the influence of the different amounts of the surfactant and the essential oil on the physicomechanical properties (droplet size, zeta potential, polydispersity index (PDI), and stability) of the emulsions. Furthermore, to our knowledge, there has been no research reported to date about the influence of an emulsions’ physicomechanical parameters on the encapsulation efficiency of essential oils and the physical properties of microcapsules such as flexibility, hardness, and diameter size.

## 2. Results and Discussion

### 2.1. Identification

**Figure 1:**
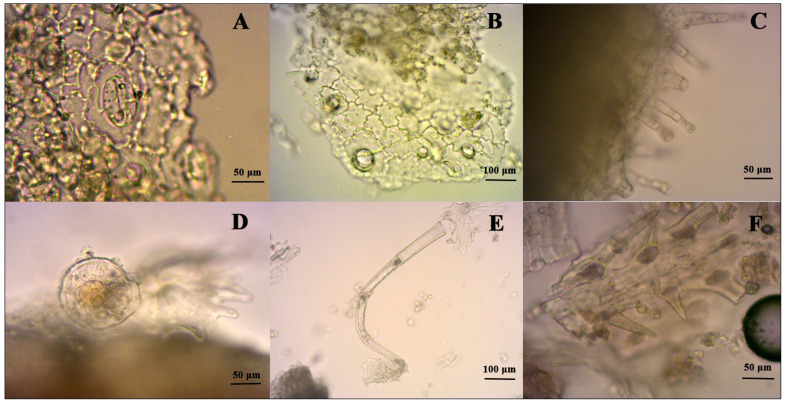
Microscopic characteristics of the oregano herb. (**A**) Stomata, magnification: 40×; (**B**) Epidermis, magnification: 20×; (**C**,**D**) Glandular trichomes, magnification: 40×; (**E**) Multicellular covering trichome, magnification: 20×; (**F**) Unicellular covering trichome, magnification: 40×.

Specific microscopic properties of the dried herb were compatible with the European pharmacopoeia [27] (see Figure 1). The powder color was green, and many diacytic stomata (A) were detected. Around the diacytic stomata, there were sinuous walled epidermis cells with broken trichome scars on the epidermis surface (B). Two types of glandular trichomes were seen clearly: one was of the lamiaceae type, with 8–16 cells on the surface (D); the other had a unicellular head with a uni-, or tricellular stalk (C). Several broken, multicellular covering hairs were seen (E). Unicellular covering hairs were short and conical (F).

**Figure 2:**
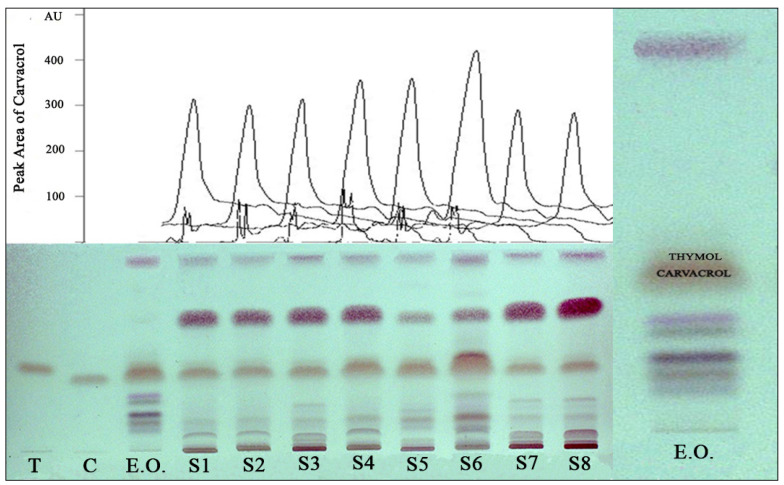
HPTLC chromatogram and peak areas of carvacrol in samples. (T: Thymol, C: Carvacrol, E.O.: Essential oil, S: Sample).

According to the European Pharmacopoeia, the chemical composition of an oregano herb has to include thymol and carvacrol [27]. On a TLC plate, both thymol and carvacrol were found in the oregano oil used in this study, though a chromatogram confirmed that carvacrol was present in much higher quantities. All of the samples had thymol and carvacrol, and among them, sample number 6 had the largest peak area of carvacrol in its structure (Figure 2).

**Figure 3:**
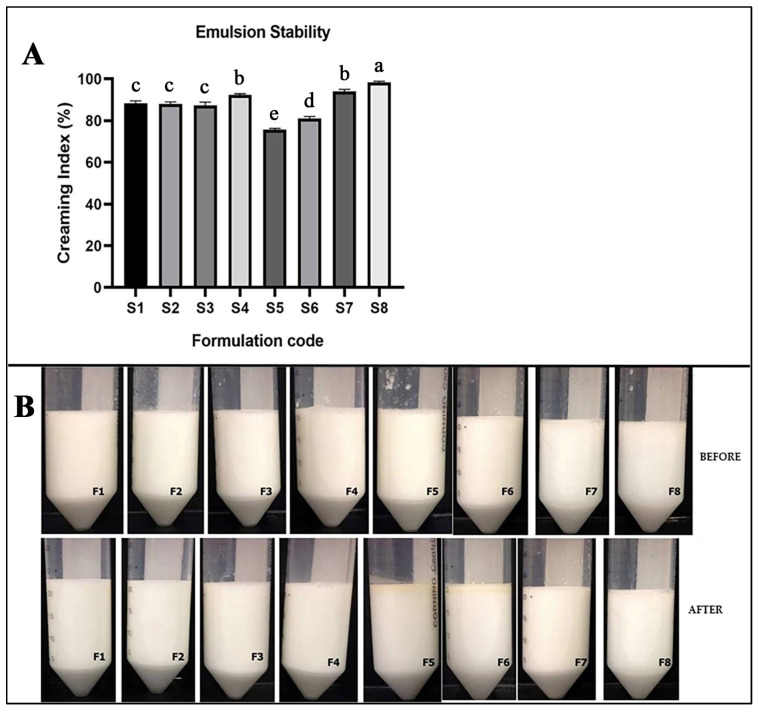
(**A**) The emulsion stability (Cl%, creaming index) of prepared emulsions (after 24 h). Data represent means ± SD, *n* = 6. Different letters in each column denote statistical difference at *p* ≤ 0.05. (**B**) Typical visual images of 8 emulsions after storage for 24 h.

The chemical composition of the hydro-distilled essential oil of oregano was determined by the GC/MS technique. Carvacrol was found to be the major component of the oregano oil used in the study, at 94.65%. In contrast, only 0.19% thymol was detected in the oil (Table 1). According to the quality criteria of the European Pharmacopoeia, the sum of thymol and carvacrol should be above 60% in oregano oil [27]. Thymol and carvacrol made up about 95% of the structure of oregano oil (Figure 3). Therefore, the chemical quality of the oil used in the study was above the specified level. Additionally, the amount of carvacrol in the structure of the samples was also examined by GC/MS. According to the obtained results, 3, 2, 2, 3, 4, 5, 2, and 2 µg/mL carvacrol were determined in samples 1–8, respectively. These amounts were consistent with the peak areas of carvacrol obtained by HPTLC, shown in Figure 2.

### 2.2. Emulsion Preparation and Characterization

Sodium alginate is the most widely used shell material for microencapsulation by extrusion [12]. The concentration of the sodium alginate solution is an important factor in formulating the emulsion, because it can influence the core materials’ degradation during the process [12]. The concentration of the sodium alginate solution was chosen according to the literature [11]. The sodium alginate solution was prepared by using a magnetic stirrer for 2 h and then the solution was left for 24 h at 25 ± 0.5 °C to get rid of the air bubbles and agglomerates of sodium alginate. The amount of the sodium alginate in all of the emulsion formulations was the same. The eight different emulsions with oregano essential oil were prepared, and the compositions of the samples are presented in Section 3. Dolca et al. performed a similar study: Rosemary oil was encapsulated by using an extrusion method and 4 g/mL of sodium alginate was used as a shell material. In that research, only the physicochemical parameters of the prepared microcapsules were analyzed; it lacked stability data about the emulsions [28].

Previous studies revealed that emulsion properties such as creaming index, z-average size of the emulsion, zeta potential, and PDI are closely associated with microencapsulation efficiency [29]. In this study, a series of emulsion properties were measured to determine how the emulsifier and essential oil ratio influences the stability and droplet size of emulsion formulations.

Creaming is a natural phenomenon in biphasic systems and it can be defined as a destabilization process [30]. Generally, this process occurs due to gravitation, which causes droplets to agglomerate, increase in size, and accumulate at the top of the system [31]. The creaming index (CI) gives a preliminary idea about emulsion stability [12]. Emulsion stability is an important factor for microencapsulation, and the emulsion should exhibit stabile characteristic properties throughout the microcapsule production process. Even if the evaluation of emulsion stability by observation of phase separation is a macroscopic and qualitative analysis, it is of great importance, especially in the encapsulation of oils, because the observation of an oily layer on the emulsion can be related to poor encapsulation efficiency of the shell material or ineffective emulsion homogenization. The creaming index is measured considering the height of the total layer and serum layer as mentioned before. The stability of the emulsions based on creaming index (%) is presented in Figure 3. The results ranged from 79.03 to 98.93%. Emulsions S4, S7, and S8 had the highest stability against creaming (CI was higher than 90%, *p* < 0.05). Moreover, the CI of the S1, S2, and S3 emulsions varied between 89.3 ± 0.34 and 90 ± 0.45% (CI was lower that 90%, *p* < 0.05). An increased amount of emulsifier (polysorbate 80) increased the stability of oregano essential oil emulsions. Moreover, the significantly lowest stability was observed in S5 and S6 formulations. The lowest CI was in emulsions with the highest amount of oregano essential oil (S5 and S6). Wang et al. achieved similar results, reporting that increasing the amount of essential oil in emulsion formulations significantly decreased the CI [32].

The mean droplet diameter (Z-averages), PDI, and zeta potential are very important indicators that describe the physical stability of the emulsions during storage [24]. The mean droplet diameter, polydispersity index (PDI), and zeta potential of the emulsions are shown in Table 2. The mean droplet diameter of all the samples was <1119.0 ± 8.8 nm. The results showed that an increasing surfactant ratio in the emulsion decreased the mean droplet size. The smallest droplet size was revealed in the S8 formulation, which contained the highest amount of polysorbate 80 in the emulsion. The main role of the surfactant is to stabilize the emulsion by reducing surface tension at the oil/water interface [33]. Only sufficient surfactants are able to dissolve oils and adjust the interfacial tension of the emulsions [33]. Thus, the oil/surfactant ratio plays a crucial role in determining the particle size and stability of the emulsions. Moreover, it is important to mention that the mean droplet diameter of the emulsions did not change significantly after storage (*p* < 0.05).

The PDI values are related to the distribution homogeneity of oil droplet size, and the closer to zero the value is, the more uniform is the distribution [34]. The PDI values ranged between 0.35 ± 0.01 and 0.51 ± 0.05. The PDI depended on the oil concentration in the emulsion. With an increase in oil concentration, the droplets were not perfectly monodispersed, and the PDI values increased. Furthermore, only the S7 and S8 formulations exhibited monomodal droplet distributions (i.e., only a single peak was observed for the particle size during storage). The increase in mean droplet size is associated with aggregation phenomena and physical destabilization of the emulsion, which in turn lead to a significant change in the PDI. Moreover, it is important to mention that according to the obtained results there were no significant differences in PDI values for all formulations after 0 and 28 days (*p* > 0.05). This is an important factor for microcapsules quality, because during the process there will be no phase separation and the PDI will not affect the microcapsules size, shape, or encapsulation efficiency [34].

Lastly, zeta potential values can be related to the emulsion stability assessment. Zeta potential is defined as the electric potential at the slipping plane that separates a mobile fluid that remains attached to the surface [35]. The magnitude of the zeta potential indicates of the degree of electrostatic repulsion between molecules or similarly charged particles in the emulsion [36]. The van der Waals force is related to the flocculating of the oil droplets in the emulsion, and due to this issue, the zeta potential becomes more negative in the emulsions [37,38]. The obtained results in terms of zeta potential immediately after the production of the emulsions varied between −0.62 ± 0.02 and −0.35 ± 0.01 mV and slightly increased (the results varied between −0.62 ± 0.04 and −0.43 ± 0.03 mV) in the S3, S5, and S6 formulations after storage for 28 days. The positive or negative value of zeta potential is related on the nature of the substances in the dispersed system [37]. The obtained results confirmed those of other authors that showed dispersions with high zeta potential (negative or positive) are electrically stabilized [37]. Lastly, the obtained results showed that zeta potential of the emulsions did not change significantly after storage (*p* < 0.05).

All emulsion formulations showed a characteristic stabilized behavior with non-ionic surfactant (polysorbate 80) and an anionic shell material (sodium alginate). Moreover, it was observed that all the emulsions were stable, which indicates that the anionic and non-ionic complex molecules were able to adsorb the oil droplets and completely coat the surface of the oil droplets.

**Figure 4:**
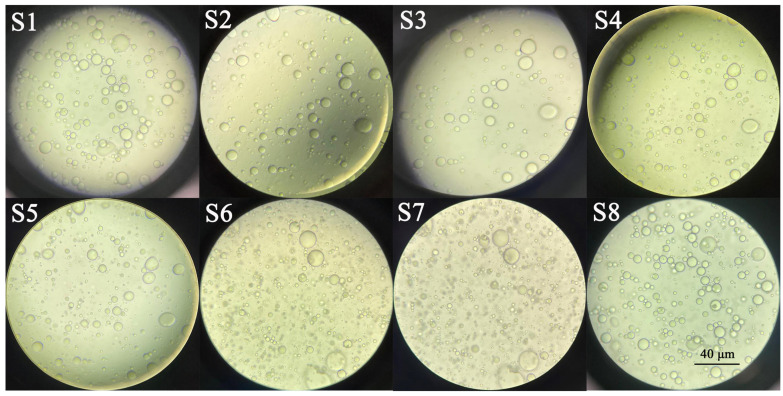
Micrographs of the oil-in-water emulsion formulations observed with an objective magnification of 40×.

Microscopic observations of the emulsions showed the presence of spherical drops (Figure 4). It was observed that emulsion droplet size significantly increased with increasing oil amount in the formulations. The obtained results could be because the smaller amount of emulsifier was not able to bind the higher amount of essential oil in the emulsions [39,40]. Garcia et al. observed a tendency of increasing average droplet size with increasing oil concentration in emulsions of basil essential oil loaded into gum arabic [41].

### 2.3. Physical Parameters of the Oregano Loaded Microcapsules

**Figure 5:**
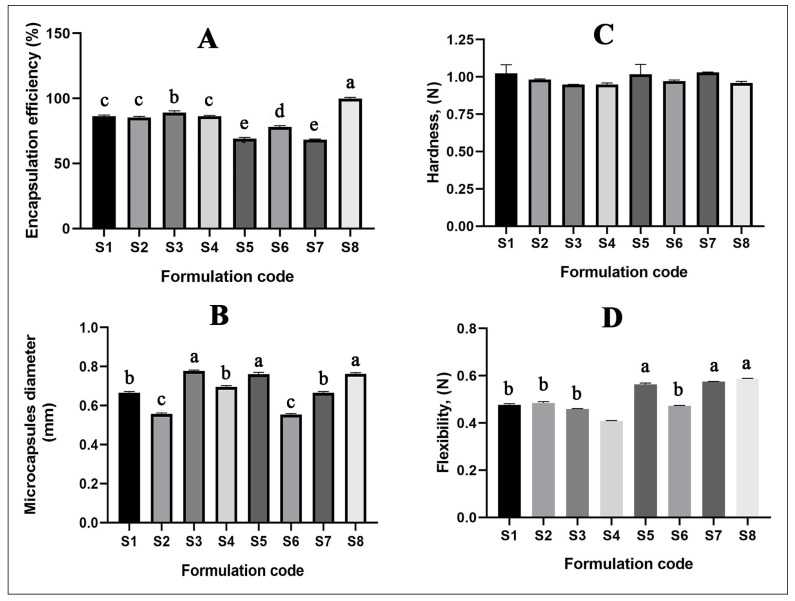
(**A**) The encapsulation efficiency of oregano essential oil-loaded microcapsules. Data represent means ± SD, *n* = 6. Different letters in each column denote a statistical difference at *p* ≤ 0.05.; (**B**) The diameter of oregano-loaded microcapsules. Different letters in each column denote a statistical difference at *p* ≤ 0.05. Data represent means ± SD, *n* = 6.; (**C**) The hardness (N) of oregano-loaded microcapsules, there was no statistical difference, *p* > 0.05. Data represent means ± SD, *n* = 6. (**D**) The flexibility (N) of oregano-loaded microcapsules. Different letters in each column denote a statistical difference at *p* ≤ 0.05 (B). Data represent means ± SD, *n* = 6.

The mechanical parameters of oregano oil-loaded microcapsules are shown in Figure 5. The results showed that encapsulation efficiency and particle size depended on the essential oil/emulsifier ratio in the emulsion (Figure 5). Encapsulation efficiency ranged from 86.3 ± 3.9% to 99.3 ± 2.4%, with the highest encapsulation efficiency found for the S8 formulation (the composition of the polysorbate 80 and oregano essential oil was 5:1) and lowest for the S7 formulation (when the ratio of polysorbate 80/oregano essential oil was 4:1). This observation suggests that the saturation capacity of the polymer to essential oil occurred at lower concentrations of essential oil. These explanations are supported by earlier work on the encapsulation of oregano essential oil in chitosan nanoparticles reported by Hosseini et al. [42]. It was represented that by increasing the initial oregano oil content from 0.1 to 0.8 g/g in chitosan nanoparticles, EE (%) decreased from 24.72 to 5.45%.

Microcapsule diameter (Figure 5) depended on the amount of shell material and emulsifier. The diameter of all tested microcapsules ranged from 0.58 to 0.79 mm, with the highest diameter found for the S3 formulation (the composition of the polysorbate 80 and oregano essential oil was 3:1) and lowest for the S6 formulation (when the ratio of polysorbate 80/oregano essential oil was 1:3). When the amount of polysorbate 80 was increased, the size of dry oregano essential oil-loaded microcapsules also decreased (*p* < 0.05). According to the literature, the surface area of a wet capsule could affect the dry microcapsule diameter [42]. Moreover, the increased surface of the wet microcapsules is mostly absorbed with increasing shell material and emulsifier amount in the formulations [42].

**Figure 6:**
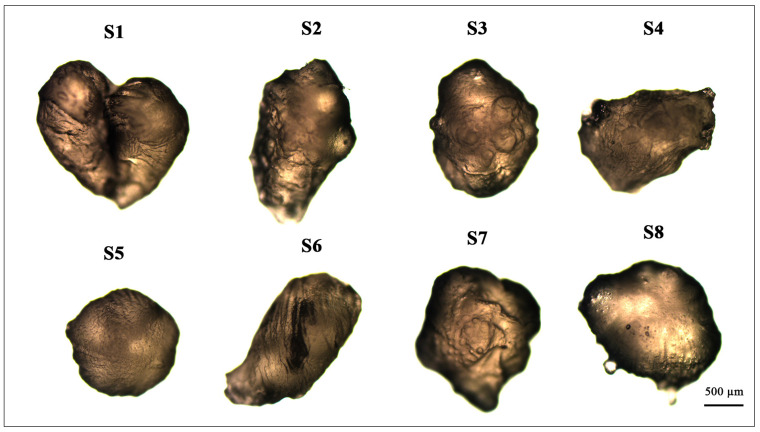
Oregano essential oil-loaded microcapsules’ (dry) surface structural changes. The codes for the microcapsules are given in Table 3. The scale bar for each microcapsule is 500 μm.

By examining the microcapsules’ surface and structure (Figure 6), the influence of emulsifier amount was determined, and the highest amount of polysorbate 80 lead to the formation of rounder capsules.

The mechanical properties were assessed using texture analysis. The force (N) needed to break (rupture and crush) the oregano essential oil-loaded microcapsules was measured (Figure 5). Tukey’s test revealed no significant difference in terms of hardness of oregano essential-loaded microcapsules. The hardness of all tested microcapsules was between 0.97 and 1.03 N, with the highest hardness found for the S5 formulation (the composition of the polysorbate 80 and oregano essential oil was 1:2) and the lowest hardness for the S4 formulation (when the ratio of polysorbate 80/oregano essential oil was 2:1). However, the essential oil/polysorbate 80 ratio did not affect the hardness of the microcapsules (*p* > 0.05). These results may be because the main effect on the hardness is the biopolymer (sodium alginate)-calcium crosslinking reactions, which could result in the creation of a rigid polymeric network [43]. As was mentioned above, the sodium alginate amount in the formulations was constant.

The flexibility of the formulated microcapsules varied between 0.407 ± 0.031 and 0.586 ± 0.046 N. Tukey’s test revealed a significant difference in terms of the flexibility of the oregano essential oil-loaded microcapsules. The S8 formulation was characterized by the highest flexibility in comparison to all other investigated combinations, the highest amount of polysorbate 80 in the formulations significantly increased flexibility of the microcapsules. The obtained results may be because the chemical nature of the shell material and surfactant complex influenced the flexibility of the microcapsules. According to the results of Draget et al., by preparing sodium alginate gel, the incorporation of more elastic, flexible materials to the matrix can permit a more rapid relaxation as a means of mechanical response to the higher deformation intensity [44]. However, the essential oil-to-polysorbate 80 ratio did not affect the flexibility of the microcapsules (*p* > 0.05).

## 3. Materials and Methods

### 3.1. Materials

#### 3.1.1. Chemicals

Oregano essential oil was used as a core material in the microencapsulation process. Standards for GC/MS and HPTLC analysis: carvacrol (>98%) and thymol (99.9%) were purchased from Sigma-Aldrich (St. Louis, MO, USA). Polysorbate 80 (Roth, Germany) was used as surfactant to stabilize the oregano emulsions. Alginic acid sodium salt was used as a shell material, and it was a gift from Deva Pharmaceutical Company (Istanbul, Turkey). Calcium chloride (Sigma-Aldrich, Munich, Germany) salt was used as a crosslinker for the production of microcapsules. Magnesium aluminometasilicate (Neusilin^®^ US2, Fuji Chemical Industries Co., Osaka, Japan) was used as an absorbent of volatile compounds. Hydrochloric acid (36%) (Sigma-Aldrich, Munich, Germany) and ethanol 99.9% (Sigma-Aldrich, Shanghai, China) were used as a solvent complex for encapsulation efficiency determination. Purified water was used throughout the experiment. Potassium dihydrogen phosphate (Supelco, Darmstadt, Germany), sodium hydroxide (Sigma- Aldrich, Munich, Germany), and sodium chloride (Sigma-Aldrich, Munich, Germany) were used to produce simulated intestinal and gastric juices. Sulfuric acid (95–97%) (Sigma-Aldrich, Munich, Germany) and vanillin (99%) (Acros Organics, Fairlawn, NJ, USA) were used for the derivatization of the HPTLC plate. Chloral hydrate (98.5%) (Acros Organics, Fairlawn, NJ, USA) was used to sharpen the images of the microscopic structures.

#### 3.1.2. Plant Material

**Figure 7:**
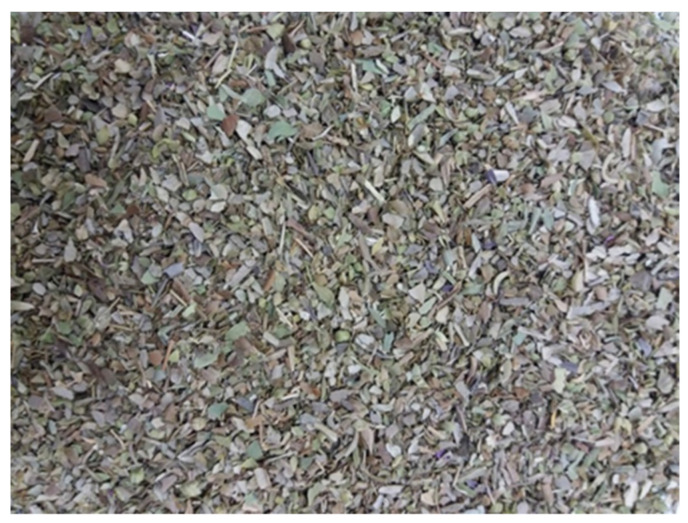
Dried oregano herb.

The dried oregano herb was purchased from Arifoglu company (İstanbul, Turkey). Due to the dried herb being in a crushed form (Figure 7), before the formulation studies, the anatomical and chemical properties of dried herb were determined to be compatible with oregano in Yeditepe University, Faculty of Pharmacy, Department of Pharmacognosy. Identification of the plant material was performed according to the requirements of the European Pharmacopoeia [27]. The tests used included a pharmacognostic investigation of microscopy, high performance thin-layer chromatography (HPTLC), and gas chromatography/mass spectrometry (GC/MS).

### 3.2. Methods

#### 3.2.1. Microscopy

Microscopic characters were examined according to the European Pharmacopoeia 8th edition, origani herba section [27]. Plant material was powdered with a grinder, then it was massed through a number 355 sieve. Powder was examined with 20× and 40× objectives and photographed under microscope (Zeiss, Axio Lab A1, Oberkochen, Germany) using a 50% chloral hydrate solution.

#### 3.2.2. Obtaining of Oregano Essential Oil by Hydro-Distillation

Two hundred grams of dried and crushed herb material was distilled with 2 L pure water for 3 h using a Clevenger type apparatus. The essential oils were dried over anhydrous sodium sulfate and stored at −20 °C until use.

#### 3.2.3. Oregano Essential Oil Emulsion Preparation

A sodium alginate solution (0.4 g/mL, *w*/*w*) was prepared by adding the required amount of sodium alginate to distilled water and stirring with a magnetic stirrer Heidolph MR 3004 (Schwabach, Germany) for 1 hour at 20 °C. The solution was used throughout the experiment for emulsion preparation as a shell material.

Emulsion was prepared as follows: 0.4 g/mL sodium alginate solution (25 mL) and the required an amount of surfactant (polysorbate 80, 0.02–0.1 g/mL) were stirred for 10 min at 20 °C. The required amount of oregano essential oil (0.02–0.06 g/mL) was added into the solution. The emulsion was stirred for 15 min at 20 °C. The compositions of the emulsions are shown in Table 3.

#### 3.2.4. Oregano Essential Oil Emulsion Stability

The stability of the emulsions to creaming was calculated according to Kibici et al. [45]. Emulsions were placed in 2.5 mL tubes and stored at 22 ± 0.5 °C for 24 h. The height of each layer was determined visually using a ruler. The creaming index (CI) was calculated as:

CI (%) = H_L_ × 100/H_E_ (1)

where H_L_ is the height of the lower serum and H_E_ is the height of the total emulsion in the cylinder.

#### 3.2.5. Emulsion Morphology

The morphology of the emulsion was examined under a light microscope Leica DM500 (Wetzlar, Genmany). The emulsion was put on a microscope slide and observed with an objective magnification of 40×. An image was taken using the camera and showed the general view of the essential oil drop.

#### 3.2.6. Characterization of Emulsion Droplet Size

The prepared emulsions’ droplet size was assessed using a Malvern brand ZS 501 Particle Sizer dynamic light scattering (DLS) instrument (Malvern, Worcestershire, UK). First, 100 μL of emulsion was diluted in purified water (1 mL). Then, the mean particle size, size distribution, and polydispersity index were expressed as an average of six trials. Zeta potential was also measured (25 °C, 90° angle) using a Malvern brand ZS 501 model Zeta-sizer instrument (DLS), (*n* = 10).

#### 3.2.7. Encapsulation of Oregano Essential Oil by Extrusion Method

Microcapsules were prepared using the extrusion method. A medical syringe (Jiangsu Zhengkang Medical Apparatus, Sanhekou, China) was used as an injector (21 gauges with diameter 0.81 mm) to prepare droplets. In total, 25 mL of alginate-oregano essential oil emulsion was injected from the needle into the crosslinker solution under stirring at 1000× rpm for 15 min. The height from the needle to the solution surface was 10 cm. Calcium chloride (0.5 g/mL) was used as the crosslinker solution. The prepared microcapsules were filtered using filter paper, washed twice with purified water, and left to dry at 20 ± 2 °C temperature for 24 h. Dried microcapsules were stored in sealed tubes until further tests.

#### 3.2.8. Physical Parameters of Microcapsules

The force and firmness of the microcapsules were analyzed by using a Texture-Analyser (TA-XT2^®^, Model 1000 R; SMS Stable Micro Systems Blackdown Rural Industries, Surrey, UK). P/100 platen was used as a probe. The force required to compress a 1 mm microcapsule (g force) was measured. The maximum force of the device was 2500 g. Analyses using a cylinder (14 mm) were performed by pressing the cylinder onto the microcapsules at a constant speed of 2 mm/s to achieve 60% compression. The strength applied on the microcapsule was progressively increased. A delay period of 10 sec was allowed between the two compressions. For one sample, 10 units of microcapsules were used. At least five replicate analyses of each sample were performed at 25 °C. Data collection and calculation were performed using the Texture Exponent 6.1.7.0 software package of the instrument. From the resulting force–time plots, hardness and fracturability were derived [46]. Microcapsule size was measured using a Digital Caliper micrometer (BGS Technic, Wermelskirchen, Germany). The diameter was determined in dried and freshly made capsules. Then, 30 units of capsules were measured, and the mean and standard deviation calculated.

#### 3.2.9. Determination of Encapsulation Efficiency

The dried oregano essential oil-loaded microcapsules (0.1–0.05 g) were dissolved in 96% ethanol (pH 1.4) (5 mL). The quantitative analysis of carvacrol was performed by the GC/MS method. The quantification was carried out by the external standard method. The calibration curves were made (R^2^ = 0.999). The encapsulation efficiency (EE) was determined by the formula:

EE (%) = qp/qt × 100(2)

where qp is the amount of the oregano essential oil in the microcapsules (mg/mL) and qt is the total oregano essential oil amount added to the microcapsules (mg/mL).

#### 3.2.10. High Performance Thin Layer Chromatography

The presence of thymol and carvacrol in the essential oil and samples were visually determined by the high performance thin layer chromatography (HPTLC) method according to the European Pharmacopoeia with minor modifications [27]. First, 5 μL of thymol (0.1 mg/mL CH_2_Cl_2_) or carvacrol (1 μL/mL CH_2_Cl_2_) standard solutions, a diluted essential oil (20 μL/mL CH_2_Cl_2_) solution, or the samples (50 mg/mL EtOH) were applied on 20 × 10 cm HPTLC glass plates coated with silica gel 60 F_254_ with an 8 mm wide band using a Limonat V automatic sample spotter (Camag, Switzerland). The plates were developed with a mixture of a toluene/ethyl acetate (99:1, *v*/*v*) solution in a twin trough chamber that was saturated for 20 min up to a distance 70 mm. The developed plates were dried with a hair dryer, then the carvacrol peak areas in the samples were determined in 270 nm using with HPTLC scanner. After scanning, plates were sprayed with 1% vanillin/sulfuric acid reagent then heated at 105 °C for 5 min on a TLC plate heater. Derivatized plate images were captured with white light with the TLC visualizer.

#### 3.2.11. GC/MS Method

The GC/MS method was performed according to the European Pharmacopoeia 8th edition, which specified the standards for carvacrol and thymol [27]. Analyses were performed using a Shimadzu GC-QP 2010 Ultra chromatography system coupled to an Electron Ionization (EI) ion source and a single quadrupole MS (Shimadzu Technologies, Kyoto, Japan). A robotic autosampler and a split/splitless injection port were used. The analytical conditions were as follows: volume injected, 1 μL; carrier gas helium, 1.22 mL/min; injector temperature, 240 °C; ion source temperature, 200 °C; interface temperature, 200 °C; split ratio, 1:20; and oven temperature ranged from 50 to 310 °C with a stepwise temperature program within the total run time of 73.00 min. The separation of analytes was carried out on a Rxi-5 ms (Restek Corporation, Bellefonte, PA, USA), capillary column (30 m long, 0.25 mm outer diameter, and 0.25 μm liquid stationary phase thickness) with a liquid stationary phase and 5% diphenyl and 95% polydimethylsiloxane with helium at a purity of 99.999% as the carrier gas in a constant flow of 1.49 mL/min. The full-scan acquisition was performed with the mass detection range set at 35–500 *m*/*z* to determine the retention times of analytes. Data acquisition and analysis were executed by LabSolution GC/MS (version 5.71) (Shimadzu Corporation, Kyoto, Japan). For the identification and quantification of the analytes, single-ion monitoring (SIM) mode was used.

#### 3.2.12. Statistical Analysis

The results were analyzed by one-way analysis of variance (ANOVA) followed by Tukey’s multiple comparison test with the software package Prism v. 5.04 (GraphPad Software Inc., La Jolla, CA, USA). The level of significance was taken as a value of *p* ≤ 0.05.

## 4. Conclusions

In this study, the effects of different amounts of emulsifier (polysorbate 80) and different emulsifier/essential oil ratios in emulsions on the prepared oregano-essential oil-loaded microcapsules parameters were evaluated. When using higher amounts of polysorbate 80 in the emulsions, higher encapsulation efficiency and smaller particle size in the microcapsules were observed. The highest encapsulation efficiency (99.3%) was observed in the F8 formulation, which had the highest amount of emulsifier. The encapsulation efficiency is the most important factor. Due to this reason, the best treatment during this research was found for the F8 formulation. Furthermore, higher concentration of the emulsifier in the formulation could also ensure the stability of the emulsion, but its amount should not be lower than 1 g (*w*/*w*). The microcapsule diameter was affected by the essential oil amount and the emulsifier amount in the emulsions. Increasing the amount of the essential oil decreased the particle diameter. However, the hardness and flexibility were not affected by the essential oil/polysorbate 80 ratio (*p* > 0.05).

In conclusion, the emulsion properties significantly influenced the prepared microcapsules’ parameters and this requires further research. Stabile emulsions with optimum compositions of oil-to-surfactant ratios will help to increase product efficiency in the pharmaceutical, cosmetic, and food industries.

## Figures and Tables

**Table 1:** Composition of the oregano essential oil used in this study.

Peak	Compounds	Area	RT (min)	%
**1**	α-Pinene	235,069	8.055	0.19
**2**	Camphene	136,820	8.824	0.11
**3**	4-Cymene	2,349,093	12.968	1.87
**4**	3-Octen-5-yne, 2,7-dimethyl-, (*E*)-	2,052,274	14.654	1.63
**5**	Thymol	240,379	24.472	0.19
**6**	Carvacrol	118,977,011	25.016	94.65
**7**	(−)-β-Caryophyllene	761,493	28.346	0.61
**8**	α-Farnesene	184,699	28.995	0.15
**9**	Longipinene epoxide	269,407	33.446	0.21

**Table 2:** The change of droplet zeta-average diameter and polydispersity index (PDI) of the formulated oil-in-water emulsions during storage. Data represent average values ± standard deviation of 10 replicates from each sample. There were no statistical differences during the storage (*p* > 0.05).

Sample Code	Parameters	Day 0	Day 3	Day 6	Day 14	Day 28
**S1**	Z-average size (nm)	806.1 ± 19.3	808.1 ± 9.3	802.5 ± 20.2	804.5 ± 17.6	806.5 ± 13.4
	PDI	0.40 ± 0.09	0.41 ± 0.07	0.42 ± 0.09	0.39 ± 0.04	0.38 ± 0.05
	Zeta potential (mV)	−0.58 ± 0.02	−0.56 ± 0.04	−0.58 ± 0.06	−0.56 ± 0.01	−0.55 ± 0.03
**S2**	Z-average size (nm)	751.2 ± 16.2	753.1 ± 15.1	753.2 ± 13.1	750.9 ± 10.3	754.0 ± 9.9
	PDI	0.42 ± 0.08	0.44 ± 0.06	0.45 ± 0.08	0.40 ± 0.07	0.43 ± 0.06
	Zeta potential (mV)	−0.49 ± 0.04	−0.49 ± 0.06	−0.50 ± 0.07	−0.48 ± 0.06	−0.49 ± 0.05
**S3**	Z-average size (nm)	643.3 ± 16.2	657.5 ± 15.8	660.3 ± 19.2	659.9 ± 12.0	658.6 ± 10.2
	PDI	0.40 ± 0.09	0.41 ± 0.05	0.41 ± 0.08	0.40 ± 0.06	0.41 ± 0.06
	Zeta potential (mV)	−0.37 ± 0.09	−0.41 ± 0.02	−0.42 ± 0.03	−0.40 ± 0.02	−0.40 ± 0.05
**S4**	Z-average size (nm)	700.1 ± 15.6	708.2 ± 5.8	709.1 ± 8.6	706.8 ± 6.5	705.1 ± 6.6
	PDI	0.4 ± 0.06	0.41 ± 0.1	0.42 ± 0.12	0.40 ± 0.11	0.41 ± 0.12
	Zeta potential (mV)	−0.50 ± 0.05	−0.50 ± 0.05	−0.50 ± 0.08	−0.49 ± 0.07	−0.50 ± 0.04
**S5**	Z-average size (nm)	755.2 ± 10.8	754.2 ± 11.2	750.3 ± 16.3	753.7 ± 10.8	755.0 ± 10.2
	PDI	0.47 ± 0.06	0.48 ± 0.08	0.48 ± 0.09	0.49 ± 0.05	0.49 ± 0.05
	Zeta potential (mV)	−0.54 ± 0.06	−0.56 ± 0.06	−0.57 ± 0.05	−0.58 ± 0.02	−0.56 ± 0.03
**S6**	Z-average size (nm)	1100.4 ± 8.3	1108.2 ± 9.8	1115.3 ± 11.8	1119.8 ± 10.2	1119.0 ± 8.8
	PDI	0.51 ± 0.05	0.48 ± 0.03	0.41 ± 0.08	0.47 ± 0.02	0.41 ± 0.05
	Zeta potential (mV)	−0.42 ± 0.03	−0.43 ± 0.04	−0.43 ± 0.07	−0.40 ± 0.05	−0.43 ± 0.03
**S7**	Z-average size (nm)	455 ± 16.5	462 ± 22.3	453.5 ± 20.4	450.9 ± 18.4	453.7 ± 15.2
	PDI	0.4 ± 0.03	0.41 ± 0.02	0.4 ± 0.02	0.4 ± 0.02	0.4 ± 0.02
	Zeta potential (mV)	−0.58 ± 0.03	−0.56 ± 0.06	−0.55 ± 0.04	−0.57 ± 0.02	−0.55 ± 0.04
**S8**	Z-average size (nm)	323.2 ± 10.2	325.5 ± 9.3	325.7 ±10.9	329.7 ±12.0	329.2 ± 9.9
	PDI	0.35 ± 0.01	0.35 ± 0.03	0.35 ± 0.05	0.35 ± 0.06	0.35 ± 0.04
	Zeta potential (mV)	−0.62 ± 0.02	−0.63 ± 0.04	−0.62 ± 0.07	−0.61 ± 0.05	−0.62 ± 0.04

**Table 3:** The compositions of the emulsions (expressed in weight-to-milliliter ratio).

Sample Code	Oregano Essential Oil (g/mL)	Polysorbate 80 (g/mL)
**S1**	0.02	0.02
**S2**	0.04	0.04
**S3**	0.02	0.06
**S4**	0.02	0.04
**S5**	0.04	0.02
**S6**	0.06	0.02
**S7**	0.02	0.08
**S8**	0.02	0.1

## Data Availability

The data presented in this study are available in this article.

## Sample Availability

Samples of the compounds are available from Juste Baranauskaite.
